# The curious case of an isolated right coronary artery aneurysm complicated by thrombosis and distal embolization

**DOI:** 10.21542/gcsp.2022.9

**Published:** 2022-06-30

**Authors:** Hussam Al Hennawi, Mohammad F. Mathbout, Katrina Bidwell, Christopher D. Nielsen

**Affiliations:** 1 Department of Internal Medicine, Jefferson Abington Hospital, Abington, PA, USA; 2 Medical University of South Carolina, Department of Cardiology, Charleston, South Carolina, USA

## Abstract

A 40-year-old male patient with no significant medical history was admitted with an inferior ST-segment elevation myocardial infarction. Primary percutaneous coronary intervention revealed a right coronary artery aneurysm, with no evidence of significant coronary disease. We support the hypothesis of aneurysmal thrombus formation with distal embolization.

## Introduction

A coronary artery aneurysm (CAA) is a localized dilation of the coronary vasculature of at least 1.5 times the diameter of an adjacent normal segment, which is a relatively uncommon finding in coronary angiography. CAA-associated ST-segment elevation myocardial infarction (STEMI) is rarely encountered in clinical practice and presents a management challenge^1^. Current guidelines lack recommendations regarding medical therapy for patients with CAA^2^. Reports indicate that two-thirds of patients presenting with CAA are empirically managed with dual antiplatelet therapy. Nevertheless, retrospective data claim that intense antithrombotic therapy is associated with better outcomes^3^. Due to the scarcity of recommendations and varied coronary anatomy, management plans remain individualized according to patient presentation, inherent CAA morphology, and complication risk.

### Case presentation

A 40-year-old male patient with a history of gout presented with acute onset of severe and persistent chest pain, which was associated with sweating, pain radiation to the left arm with a tingling sensation, and concurrent acute-onset diarrhea. Emergency medical service was called, troponin peak was 45,909 (<=27 ng/L normal) with EKG readings consistent with ST-elevation myocardial infarction (STEMI). The patient was then transferred to the catheterization lab for further management.

**Figure 1.**
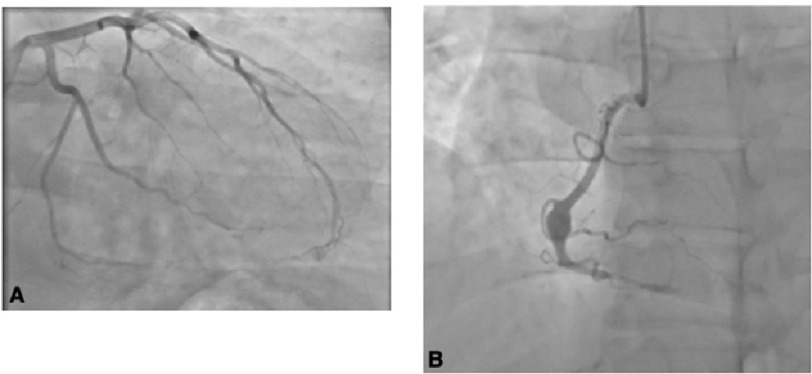
Coronary angiography showing normal LMCA and left main branches (A). Aneurysmal Mid-RCA with thrombotic occlusion of the distal vessel (B).

**Figure 2.**
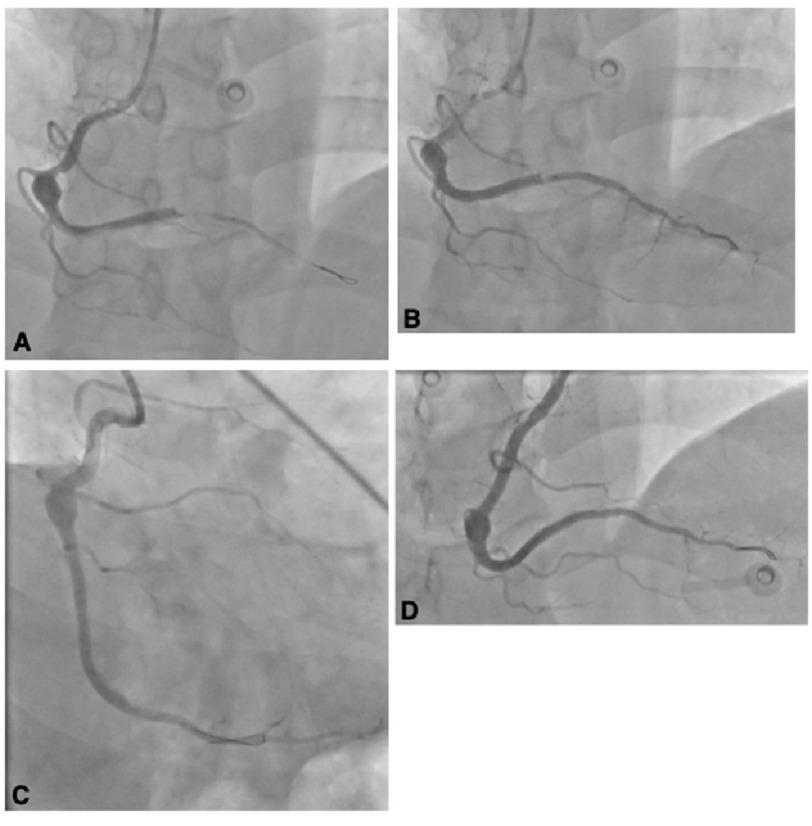
RCA angiogram. (A) RCA wiring with a Fielder FC wire. (B) PDA flow restoration following ballooning. (C) No visible posterolateral flow. D: Distal RCA following stent deployment demonstrating no residual stenosis.

Transthoracic echocardiography (TTE) showed mild left ventricular (LV) dilatation with hypokinesia of the inferolateral wall, with an ejection fraction of 69%. A coronary angiogram demonstrated normal left main coronary artery, left anterior descending (LAD), and left circumflex (Figure 1A), an isolated mid-right coronary artery (RCA) aneurysm, and mid-distal thrombotic occlusion of the RCA just proximal to takeoff of the posterior descending artery (PDA) branch (Figure 2B). The patient underwent percutaneous intervention in which the RCA was wired with some difficulty using Fielder FC wire (Figure 2A). The occluded area was ballooned with a 2.0 mm balloon with restoration of flow into the PDA (Figure 2B). Posterolateral branches were not observed (Figure 2C). The distal occlusion was stented with a 2.5 × 18 mm Resolute Onyx (drug-eluting) stent. A proximal large diameter segment with residual stenosis was stented with a 3.5 mm × 8 mm Resolute Onyx (drug-eluting) (Figure 2D). Postoperative angiographic results demonstrated 0% residual stenosis with no evidence of posterolateral branches in multiple views. The patient had an uneventful course of treatment with no postoperative complications and was discharged on aspirin and Plavix dual antiplatelet therapy (DAPT) for one year.

## Discussion

CAA has a 0.3−5.3% incidence rate, with a mean incidence of 1.65% from the pooled analysis^3^. Men tend to be more predisposed than women with an underlying atherosclerotic coronary artery disease (CAD) deemed the most common risk of developing CAA in the adult population, while Kawasaki disease is in the pediatric population. Notably, growing adults with late manifestations of Kawasaki disease might also have the propensity to develop CAA. Other risk factors include connective tissue disease and infectious diseases. In 2009, Jha et al. demonstrated a modest RCA propensity for aneurysmal formation over the LAD (19 RCA *vs.* 10 LAD) with no apparent underlying reason^5^. Although CAA is most commonly an incidental finding, this can be complicated by thrombosis and distant embolism. Aneurysmal dilatation is associated with stagnant coronary blood flow, predisposing patients to the development of acute thrombus formation, presenting as angina, ischemia, or STEMI. Coronary angiography plays a vital role in the diagnosis of CAA. Cardiac intravascular ultrasound can be a valuable tool in the characterization of luminal components of aneurysms and can differentiate between true and pseudo-aneurysmal dilatations^4^.

To date, there is a lack of consensus regarding CAA management. Nevertheless, guideline-directed medical management remains the treatment of choice for CAA associated with a background of atherosclerotic CAD. Should thrombosis/embolism raise any concern, DAPT with anticoagulants should be indicated, similar to the present case^5^. This decision should be balanced by considering the patient’s bleeding risks. Rivaroxaban with DAPT is a better alternative than vitamin K antagonists and DAPT in terms of bleeding risk, as PIONEER AF-PCI demonstrated a lower risk of bleeding in the former. Similar to CAD, matrix metalloproteinase and inflammatory cytokines have also been linked to CAA, suggesting the role of statin therapy in halting aneurysmal progression^6^. Surgical modalities may be indicated in cases where a large saccular aneurysm is identified (>10 mm), which tends to present a risk of rupture. Modalities include aneurysmectomy with or without coronary bypass grafting and aneurysmal ligation/resection.

**Figure 3.**
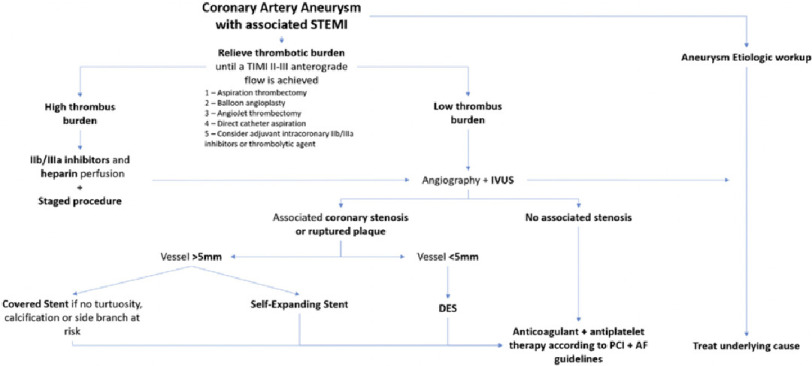
Treatment algorithm for ST-segment elevation acute myocardial infarction (STEMI) caused by coronary artery aneurysm. The primary objective should be a return of anterograde circulation and thrombus burden reduction. Intravascular ultrasound (IVUS) may play a role in understanding the base mechanism, vessel sizing and optimize stent implantation. AF = atrial fibrillation; DES = drug-eluting stent; PCI = percutaneous coronary intervention; TIMI = Thrombolysis In Myocardial Infarction. Reproduced under CC BY license from^2^.

Our case is of further interest as there was no associated CAD with RCA aneurysmal finding. This finding made the theory of aneurysmal thrombus formation with distant embolization sound plausible. The management of patients with CAA-associated STEMI poses a challenge and can be associated with high-risk complications. Despite the successful revascularization outcome in this case, revascularization of an aneurysmal coronary artery vessel has a relatively low success rate and carries >15% risk rate of stent re-thrombosis at one year^7^. Therefore, revascularization of an aneurysmal culprit vessel should focus on anterograde reperfusion as a primary objective, followed by stent implantation. The current percutaneous recommendations for CAA-associated STEMI are presented in (Figure 3)^2^. However, it is important to understand the nature of CAA development. In the case of stenotic aneurysms associated with Kawasaki disease, lesions were shown to be highly calcified which may require a different management strategy from typical atherosclerotic lesions including rotational atherectomy since the use of high balloon pressure with angioplasty can lead to aneurysmal formation^8–10^.

### What have we learned?

• Coronary artery aneurysms have a predilection to develop in right coronary artery affecting 40–70% of cases and tend to be associated with multi-vessel atherosclerotic coronary artery disease.
• Medical management still constitutes the preferred management for coronary artery aneurysms in the setting of atherosclerosis.
• Further studies are needed to investigate the optimal management of artery aneurysm.

References1.
KawsaraA
Núñez GilI
AlqahtamiF
MorelandJ
RihalC
AlkhouliM
2018Management of coronary artery aneurysmsJ Am Coll Cardiol Intv1112111223doi: 10.1016/j.jcin.2018.02.0412.
Ponte MonteiroJ
Flores-UmanzorE
BrugalettaS
2020STEMI with a massive coronary aneurysm: a rare finding with a management dilemmaJACC Case Rep23477479Published 2020 Mar 18. doi: 10.1016/j.jaccas.2020.01.01534317268PMC83116903.
Núñez-GilIJ
TerolB
FeltesG
Nombela-FrancoL
SalinasP
EscanedJ
Jiménez-QuevedoP
GonzaloN
VivasD
BautistaD
MacayaC
Fernández-OrtizA
2018Coronary aneurysms in the acute patient: incidence, characterization and long-term management resultsCardiovasc Revascularization Med458959610.1016/j.carrev.2017.12.003292761764.
HartnellGG
ParnellBM
PridieRB
1985Coronary artery ectasia, Its prevalence and clinical significance in 4993 patientsBritish Heart Journal54392395doi: 10.1136/hrt.54.4.3924052280PMC4819175.
GeJ
LiuF
KearneyP
GörgeG
HaudeM
BaumgartD
AshryM
ErbelR
1995Intravascular ultrasound approach to the diagnosis of coronary artery aneurysmsAmerican Heart Journal130765771doi: 10.1016/0002-8703(95)90075-675725846.
TiryakiogluO
DemirtasS
AriH
TiryakiogluSK
HuysalK
SelimogluO
OzyaziciogluA
2009Magnesium sulphate and amiodarone prophylaxis for prevention of postoperative arrhythmia in coronary by-pass operationsJ Cardiothorac Surg48Published 2009 Feb 20. doi: 10.1186/1749-8090-4-819232084PMC26499247.
DemopoulosVP
OlympiosCD
FakiolasCN
PissimissisEG
EconomidesNM
AdamopoulouE
FoussasSG
CokkinosDV
1997The natural history of aneurysmal coronary artery diseaseHeart78136141doi: 10.1161/01.CIR.93.9.17099326986PMC4848928.
InoT
AkimotoK
OhkuboM
NishimotoK
YabutaK
TakayaJ
YamaguchiH
1996Application of percutaneous transluminal coronary angioplasty to coronary arterial stenosis in Kawasaki diseaseCirculation93917091715doi: 10.1161/01.CIR.93.9.170986538779.
UenoT
IshiiM
AkagiT
BabaK
HaradaK
HamaokaK
KatoH
TsudaE
UemuraS
SajiT
OgawaS
EchigoS
YamaguchiT
2001Guidelines for catheter intervention in coronary artery lesion in Kawasaki diseasePediatr Int435558562doi: 10.1046/j.1442-200X.2001.01464.x1173772810.
TsudaE
MiyazakiS
YamadaO
TakamuroM
TakekawaT
EchigoS
2006Percutaneoustransluminal coronary rotational atherectomy for localized stenosis caused by Kawasaki diseasePediatric Cardiology274447453doi: 10.1007/s00246-006-1276-516830078
